# Syntaxin-3 Binds and Regulates Both R- and L-Type Calcium Channels in Insulin-Secreting INS-1 832/13 Cells

**DOI:** 10.1371/journal.pone.0147862

**Published:** 2016-02-05

**Authors:** Li Xie, Subhankar Dolai, Youhou Kang, Tao Liang, Huanli Xie, Tairan Qin, Lu Yang, Liangyi Chen, Herbert Y. Gaisano

**Affiliations:** 1 Department of Medicine, Faculty of Medicine, University of Toronto, Toronto, ON, Canada; 2 Institute of Molecular Medicine, Peking University, Beijing, China; Indiana University School of Medicine, UNITED STATES

## Abstract

Syntaxin (Syn)-1A mediates exocytosis of predocked insulin-containing secretory granules (SGs) during first-phase glucose-stimulated insulin secretion (GSIS) in part via its interaction with plasma membrane (PM)-bound L-type voltage-gated calcium channels (Ca_v_). In contrast, Syn-3 mediates exocytosis of newcomer SGs that accounts for second-phase GSIS. We now hypothesize that the newcomer SG Syn-3 preferentially binds and modulates R-type Ca_v_ opening, which was postulated to mediate second-phase GSIS. Indeed, glucose-stimulation of pancreatic islet β-cell line INS-1 induced a predominant increase in interaction between Syn-3 and Ca_v_α1 pore-forming subunits of R-type Ca_v_2.3 and to lesser extent L-type Ca_v_s, while confirming the preferential interactions between Syn-1A with L-type (Ca_v_1.2, Ca_v_1.3) Ca_v_s. Consistently, direct binding studies employing heterologous HEK cells confirmed that Syn-3 preferentially binds Ca_v_2.3, whereas Syn-1A prefers L-type Ca_v_s. We then used siRNA knockdown (KD) of Syn-3 in INS-1 to study the endogenous modulatory actions of Syn-3 on Ca_v_ channels. Syn-3 KD enhanced Ca^2+^ currents by 46% attributed mostly to R- and L-type Ca_v_s. Interestingly, while the transmembrane domain of Syn-1A is the putative functional domain modulating Ca_v_ activity, it is the cytoplasmic domain of Syn-3 that appears to modulate Ca_v_ activity. We conclude that Syn-3 may mimic Syn-1A in the ability to bind and modulate Ca_v_s, but preferring Ca_v_2.3 to perhaps participate in triggering fusion of newcomer insulin SGs during second-phase GSIS.

## Introduction

Soluble *N*-ethylmaleimide-sensitive factor attachment protein receptor (SNARE) proteins, including target- (t-) membrane SNAREs (Syntaxins [Syn]) and synaptosomal-associated proteins of 25 kDa (SNAP25) and vesicle-associated membrane proteins (VAMPs), are the fundamental components of the exocytotic machinery required for the docking and fusion of secretory granules (SGs) with the plasma membrane (PM), which have been well studied in neurons [1, 2] and neuroendocrine cells, particularly pancreatic islet β-cells [3–5]. t-SNAREs Syn-1A and SNAP25 through their interactions with PM-bound voltage-gated calcium channels (Ca_v_), L-type in β-cells and N-type in neurons, position the predocked SGs to the site of maximum Ca^2+^ influx for efficient exocytosis [6–12].

Ca_v_s regulate secretion in neurons and β-cells [13, 14]. Ca_v_α1 pore-forming subunits, Ca_v_1 and Ca_v_2, exist as heteromeric complexes by association with auxiliary subunits, β and α_2_δ subunits, which mediate trafficking of Ca_v_s to the PM and fine-tune their biophysical properties [13, 14]. In β-cells, L-type Ca_v_1s (Ca_v_1.2 is abundant in rodents; Ca_v_1.3 is abundant in human) [15, 16], are believed to effect first-phase GSIS by acting on the readily releasable pool (RRP) of predocked SGs [17–20]. Genetic deletion of R-type/Ca_v_2.3 suppressed only the second-phase GSIS from the mouse islets; and did not affect the early component of depolarization-induced exocytosis (corresponding to the RRP) in the β-cells [21, 22], leaving intact the late component, referred to as SG mobilization from the reserve pool, which corresponds to the newcomer SGs. This led us and others to hypothesize that predocked SGs mediating a major portion of first-phase GSIS and newcomer SGs accounting for all of second-phase GSIS are respectively mediated by L- and R-type Ca_v_s.

Of the four Syns that mediate exocytosis, Syn-1A, Syn-2 and Syn-4 are present and localized predominantly to the β-cell PM, whereas Syn-3 is more abundant in SGs [5, 23–25]. Genetic deletion of Syn-1A in mice blunted first-phase GSIS, which was attributed to loss of ability of predocked insulin SGs to undergo exocytosis, without perturbation of recruitment and fusion of newcomer SGs [5]. Syn-1A binding and inhibition of L-type Ca_v_ [9, 10] was demonstrated to be via the two highly conserved cysteines, Cys271 and Cys272 at its transmembrane domain [26–28]. In contrast, depletion of endogenous Syn-3 by RNA interference (RNAi) in INS-1 cells inhibited GSIS by impairing the recruitment and fusion of newcomer SGs affecting predominantly the second-phase GSIS, without affecting predocked SGs [23]. Overexpression of Syn-3 was reported to also inhibit β-cell L-type Ca_v_ [9]. However, it remains unclear whether endogenous Syn-3 modulates Ca_v_ channels in β-cells. Furthermore, the putative Ca_v_-interacting transmembrane cysteine residues in Syn-1A are not conserved in Syn-3. Therefore, more work is required to clarify which β-cell Ca_v_s Syn-3 acts on and the putative Ca_v_-binding domain within Syn-3.

In this work, we assessed the endogenous function of Syn-3 on β-cell Ca_v_ activity by siRNA depletion and provide detailed biochemical and functional evidence for the interactions between endogenous Syn-3 and R-type (Ca_v_2.3) and to lesser degree also L-type (Ca_v_1.2 and Ca_v_1.3) Ca_v_s.

## Methods

### Cell Culture

INS-1 832/13 cells and HEK293 cells lines were cultured as previously reported [29, 30]. INS-1 832/13 cell line (herein called INS-1) was a gift from Christopher Newgard (Duke University, Durham, North Carolina) [31]. Syn-3 siRNA/mCherry plasmid (Dharmacon, Chicago, IL, USA) used here we previously reported to efficiently knockdown (KD) Syn-3 expression in INS-1 cells [23]. A mCherry plasmid was used as control [23]. After the cells were transfected with these plasmids for 48 h, cellular entry of the plasmids was confirmed by the mCherry expression observed by epifluorescence imaging. These mCherry-expressing cells were subjected to electrophysiology and TIRF imaging studies.

### Immunoprecipitation

This was performed on INS-1 cells as previously reported [30, 32]. INS-1 cells at 80%–85% confluence were washed with PBS (37°C) and incubated for 30 min at 37°C in Krebs–Ringer HEPES buffer (KRB, in mM: 125 NaCl, 5.6 KCl, 1.28 CaCl_2_, 5 Na_2_CO_3_, 25 HEPES, pH 7.4. with 0.1% BSA) containing basal 0.8 mM glucose concentration. Cells intended for stimulation were preincubated for 30 min with 0.8 mM glucose, and then stimulated with 16.7 mM glucose plus 10 nM glucagon-like peptide (GLP)-1 for 30 min. 1 mg protein extract of cell lysates were used for each condition. Immunoprecipitation (IP) was with 2 μg Syn-1A or Syn-3 antibodies or pre-immune IgG (as control). Precipitated proteins were immunodecorated and identified using the indicated primary antibodies, which include anti-Ca_v_1.2, -Ca_v_1.3, -Ca_v_2.3, -Ca_v_2.2, -Ca_v_α_2_δ-1, -Ca_v_β3, -SNAP25, -Syn-1A and–Syn-3. All Ca_v_ subunits antibodies are from Alomone Labs (Jerusalem, Israel), and SNAP25, Syn-1A and Syn-3 are from SySy (Goettingen, Germany); the specificity of these antibodies was well characterized by these companies, which been used broadly. IP experiments on HEK293 cells transfected with Syn-1A, Syn-3, Ca_v_1.3 or Ca_v_2.3, were similar to those performed on INS-1 cells.

### In Vitro Binding Assay and Western Blotting

In vitro binding assays were performed according to the method we previously described [30, 32, 33], which also showed the specificity of the SNARE protein antibodies. Briefly, GST-Syn-1A (cytoplasmic domain a.a. 1–265), GST-Syn-3 (cytoplasmic domain a.a. 1–263) and GST (as control, 300 pM protein each, all bound to glutathione agarose beads) were incubated with HEK293 cell lysate at 4°C for 2 h with constant agitation. The beads were then washed three times with washing buffer (in mM: 20 HEPES (pH 7.4), 150 KOAC, 1 EDTA, 1 MgCl_2_; with 5% glycerol and 0.1% Triton X-100). Precipitated proteins were separated on 10% SDS-PAGE and identified with anti-Ca_v_1.3 or -Ca_v_2.3 antibody (1:200).

All of the Western blot bands were quantified using image J software (http://rsb.info.nih.gov/ij). We employed two approaches to quantify the ‘input control’ and ‘co-immunoprecipitated (co-IPed)’ blots. For quantification of ‘input control’ bands, we considered maximum intense band for each protein from each experiment as 100 and expressed other bands for that particular protein as % of maximum. For quantification of ‘co-IPed’ blots, we measured intensities of both ‘co-IPed’ and ‘input control’ bands that are processed in parallel. The intensity of ‘co-IPed’ band was then calculated as a ratio to the corresponding ‘input control’ band intensity and multiplied by 5 (as ‘input control’ is 5% of total protein used for IP) and expressed as ‘percentage of recovery’.

### Electrophysiology

Recording pipettes were pulled from 1.5-mm borosilicate glass capillary tubes using a programmable micropipette puller. Pipettes were heat polished to a tip resistance ranging from 2 to 3 MOhm when filled with the intracellular solution. For measurement of Ca_v_ currents, barium was used as charge carrier. Pipettes were filled with the buffer containing (in mM): 120 CsCl, 20 tetraethylammonium chloride, 5 EGTA, 5 MgATP and 5 HEPES (pH 7.2 with CsOH). The external solution contained (in mM): 100 NaCl, 20 BaCl_2_, 20 tetraethylammonium chloride, 4 CsCl, 1 MgCl_2_, 10 glucose and 5 HEPES (pH 7.4 with NaOH). L-type Ca_v_ inhibitor nifedipine (10 μM), R-type Ca_v_ inhibitor SNX482 (400 nM) and N-type Ca_v_ inhibitor ω-Conotoxin GVIA (100 nM) are all from Alomone labs (Jerusalem, Israel). Cells were held at −70 mV for 2 min after formation of whole-cell mode, and currents elicited by stepped 300 or 500 milliseconds depolarizations in 10 mV increments. Recordings were conducted using an EPC10 patch clamp amplifier equipped with Pulse and X-Chart software programs (HEKA Electronik, Lambrecht, Germany).

### Statistical Analysis

Data are presented as mean±SEM. Statistical significance was evaluated by Student’s t test or Mann-Whitney rank sum test (SigmaStat 3.11. Systat Software Inc., Chicago, IL, USA) and considered significant when P<0.05.

## Results

### Syn-3 Binds to Distinct β-cell Ca_v_s than Syn-1A

**Fig 1:**
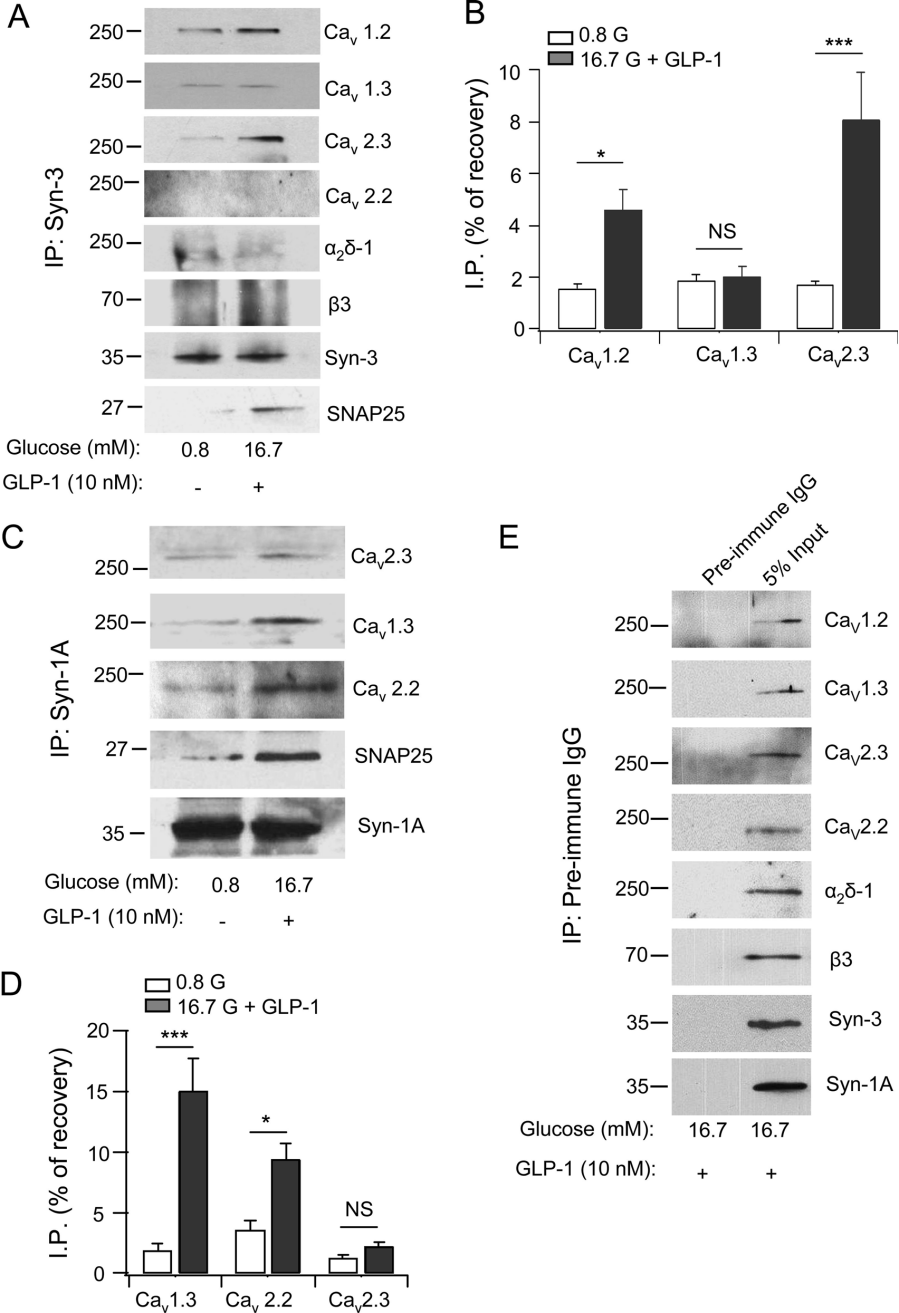
Syn-3 co-immunoprecipitates (IP) distinct Ca_v_s than Syn-1A in INS-1 cells. Syn-3 (A) and Syn-1A (C) interactions with the indicated Ca_v_α1 subunits (Ca_v_1.2, Ca_v_1.3, Ca_v_2.3 and Ca_v_2.2) and auxiliary subunits (α_2_δ-1 and β3) and SNAP25 in INS-1 cells. Densitometric analysis of Syn-3 co-IP (B) and Syn-1A co-IP (D), expressed as percent recovery of total lysate inputs (which showed equal protein loading in S1 Fig), shows that high glucose (16.7 mM) plus GLP-1 (10 nM) increased the association of these syntaxins with the respective Ca_v_s. Values are means±SEM, n = 3. *p<0.05, ***p<0.001, NS: not significant. As control (E) shows representative blots from five separate co-IP experiments with pre-immune IgG, which did not bring down syntaxins or Ca_v_s (*left* lanes). *Righ*t lanes show the input lysates. All five experiments probed for the Ca_v_ α subunits and α_2_δ-1, whereas β3, Syn-3 and Syn-1A were probed on two blots from separate experiments.

We postulated that Syn-3’s actions on Ca_v_ channels [9] may be by direct physical binding to Ca_v_ subunits as had been demonstrated for Syn-1A [11, 12]. This was assessed with Syn-3 antibody co-immunoprecipitation of INS-1 at basal (0.8 mM glucose) and maximal stimulated conditions (16.7 mM glucose plus 10 nM GLP-1). Fig 1A and 1B show that Syn-3 co-precipitated Ca_v_α1 pore-forming subunits including Ca_v_1.2, Ca_v_1.3 and Ca_v_2.3, but not Ca_v_2.2. Syn-3 also brought down small amounts of auxiliary subunits α_2_δ-1 and β3. Remarkably, GLP-1/high glucose stimulation caused a large increase in the amount of Ca_v_2.3 co-precipitated (from 1.7% to 8.1%, a 4.7 fold increase; p<0.001), a more moderate increase in Ca_v_1.2 co-precipitated (from 1.6% to 4.6%, a 2.9 fold increase; p<0.05), and no significant increase in Ca_v_1.3 co-precipitated (1.9% to 2.0%). There was no increase in the levels of co-precipitated auxiliary subunits α_2_δ-1 and β3; and there was no detectable Ca_v_2.2 brought down. There was also an expected large increase in the amount of SNAP25 co-precipitated (from 0.9% to 7.5%, p<0.001). These results indicate a preferential formation of the Syn-3/Ca_v_2.3 α1 complex, and the more moderate formation of the Syn-3/Ca_v_1.2 complex could explain our previous report on why Syn-3 overexpression also affected L-type Ca_v_ current [9].

Syn-1A antibody co-precipitated Ca_v_1.3, Ca_v_2.2 and Ca_v_2.3 α1 subunits (Fig 1C). However, only the amounts of Ca_v_1.3 (from 1.9% at basal to 15% when stimulated, a 7.8 fold increase; p<0.001; N = 3; Fig 1C and 1D) and Ca_v_2.2 (from 3.6% to 9.4%; p<0.05; N = 3; Fig 1C and 1D) to lesser degree increased with stimulation, whereas there was no further increase in the amount of Ca_v_2.3 co-precipitated. SNAP25 co-precipitated increased from 1.25% to 6.3% (p<0.05; N = 3) after stimulation. The latter results confirm the previous hypothesis that Syn-1A preferentially associates with L-type Ca_v_ to form a complex exictosomes, which is functionally important in mediating exocytosis of predocked insulin SG [6, 7]. While our results support that Syn-1A could also bind R-type Ca_v_ [34], this complex is perhaps less important in β-cell or at least subordinate to the more abundant Syn-3-R-type Ca_v_ complex that formed. Syn-1A is known to also bind N-type Ca_v_ in neurons [35] that is also found in INS-1 [34], albeit less abundant, thus presumably less important in β-cell. As control, at maximal stimulation with high glucose plus GLP-1, preimmune IgG did not pull down the syntaxins or any of the Ca_v_ subunits (Fig 1E). S1 Fig shows the corresponding input proteins with the Syn-3 or Syn-1A IP experiments in Fig 1 in both control and stimulated INS-1 cells.

### Syn-3 Preferentially Binds Ca_v_2.3, whereas Syn-1A Preferentially Binds Ca_v_1.2 and Ca_v_1.3

The co-IP studies of endogenously interacting proteins (Fig 1) showed a preference of Syn-3 for R-type/Ca_v_2.3 over L-type Ca_v_s. We therefore next critically assessed whether Syn-3 vs Syn-1A do indeed preferentially bind R- and L-type Ca_v_s, respectively, by employing the HEK cell model. HEK cells, devoid of endogenous Ca_v_s and SNARE proteins, allow these proteins to be individually exogenously expressed presumably in their native conformations, unperturbed by any other proteins that might affect them or their interactions as may be the case using native β-cells (Fig 1).

**Fig 2:**
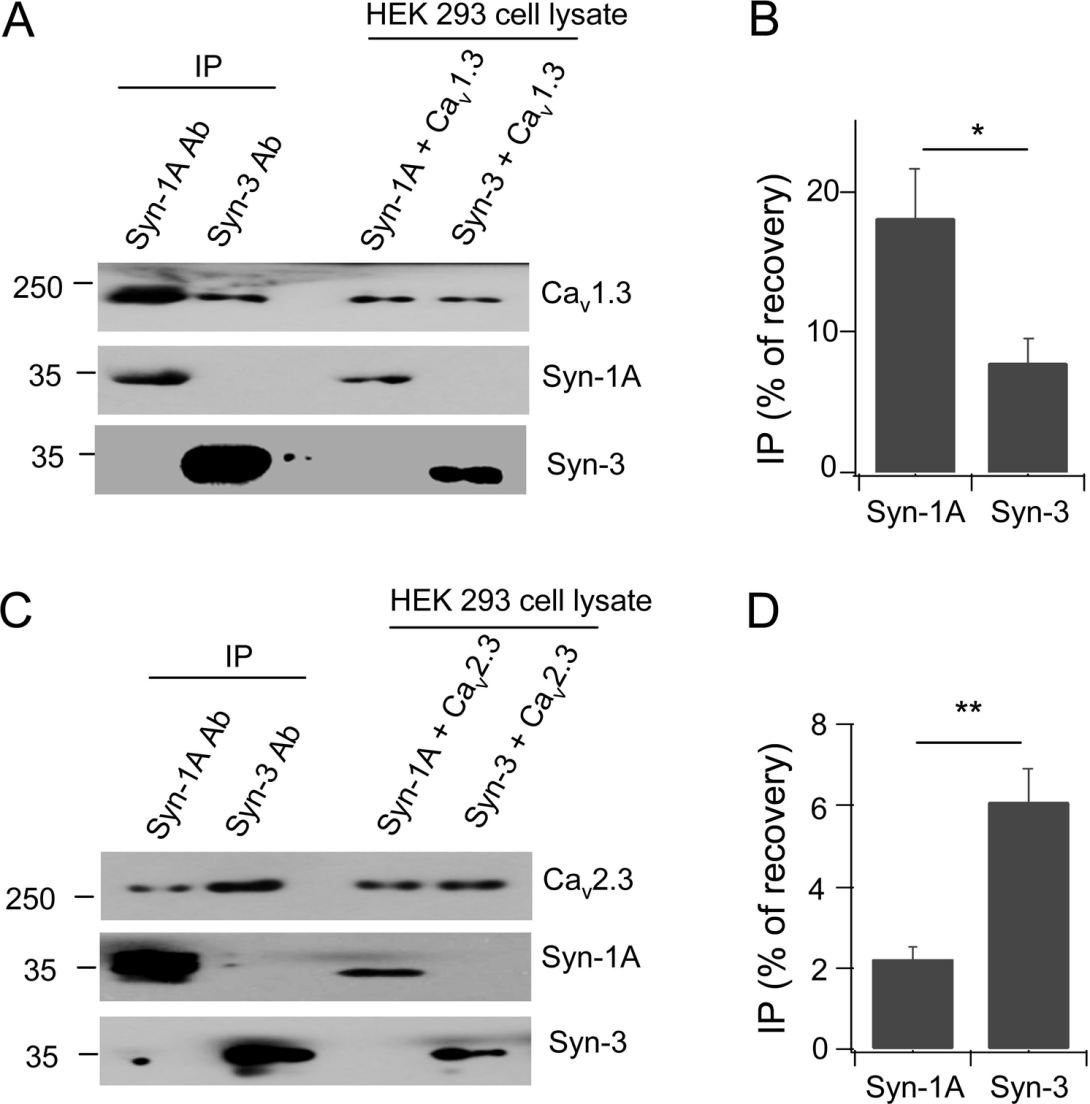
Syn-3 preferentially binds Ca_v_2.3 while Syn-1A preferentially binds Ca_v_1.3. Representative blots show HEK293 cells co-transfected with Ca_v_1.3 (A) or Ca_v_2.3 (C) with either Syn-1A or Syn-3, then subjected to co-IP with anti-Syn-1A or Syn-3 antibody. Co-precipitated proteins were identified with the indicated antibodies. Densitometric analysis of the co-precipitated Ca_v_1.3 (B) or Ca_v_2.3 (D), expressed as percent recovery of total lysate inputs. Values are means±SEM, n = 3; *p<0.05, **p<0.01.

Immunoprecipitation experiments were conducted in HEK cells expressing only Syn-3 or Syn-1A with Ca_v_1.3 or Ca_v_2.3 α1 subunits. When calculated as the percentage of protein recovery from total lysate input, Syn-1A co-precipitated Ca_v_1.3 with an average (N = 3) of 18.1±3.5% versus Syn-3 of 7.8±1.8%, which is 2.3 times higher (Fig 2A and 2B). In contrast, Syn-3 antibody co-precipitated more Ca_v_2.3 (6.1±0.8%) than Syn-1A antibody (2.2±0.3%), which is 2.8 times higher (Fig 2C and 2D). Therefore, while there is some promiscuity in the binding of Syn-1A and Syn-3 for these Ca_v_s, Syn-1A preferentially binds Ca_v_1.3 and Syn-3 preferentially binds Ca_v_2.3. This is consistent with the current thinking that while the Syn-1A-Ca_v_1.3 complex targets the PM sites of maximal Ca^2+^ influx to where predocked insulin SGs exocytose [6, 7], we further postulate that the Syn-3-Ca_v_2.3 complex likely targets the PM sites of Ca^2+^ influx where exocytosis of newcomer insulin SGs [23] would likely occur. This thinking is also consistent with the role of Ca_v_2.3 in second-phase GSIS [21].

### Depletion of Endogenous Syn-3 Selectively Enhances Ca_v_ Channels Activity

**Fig 3:**
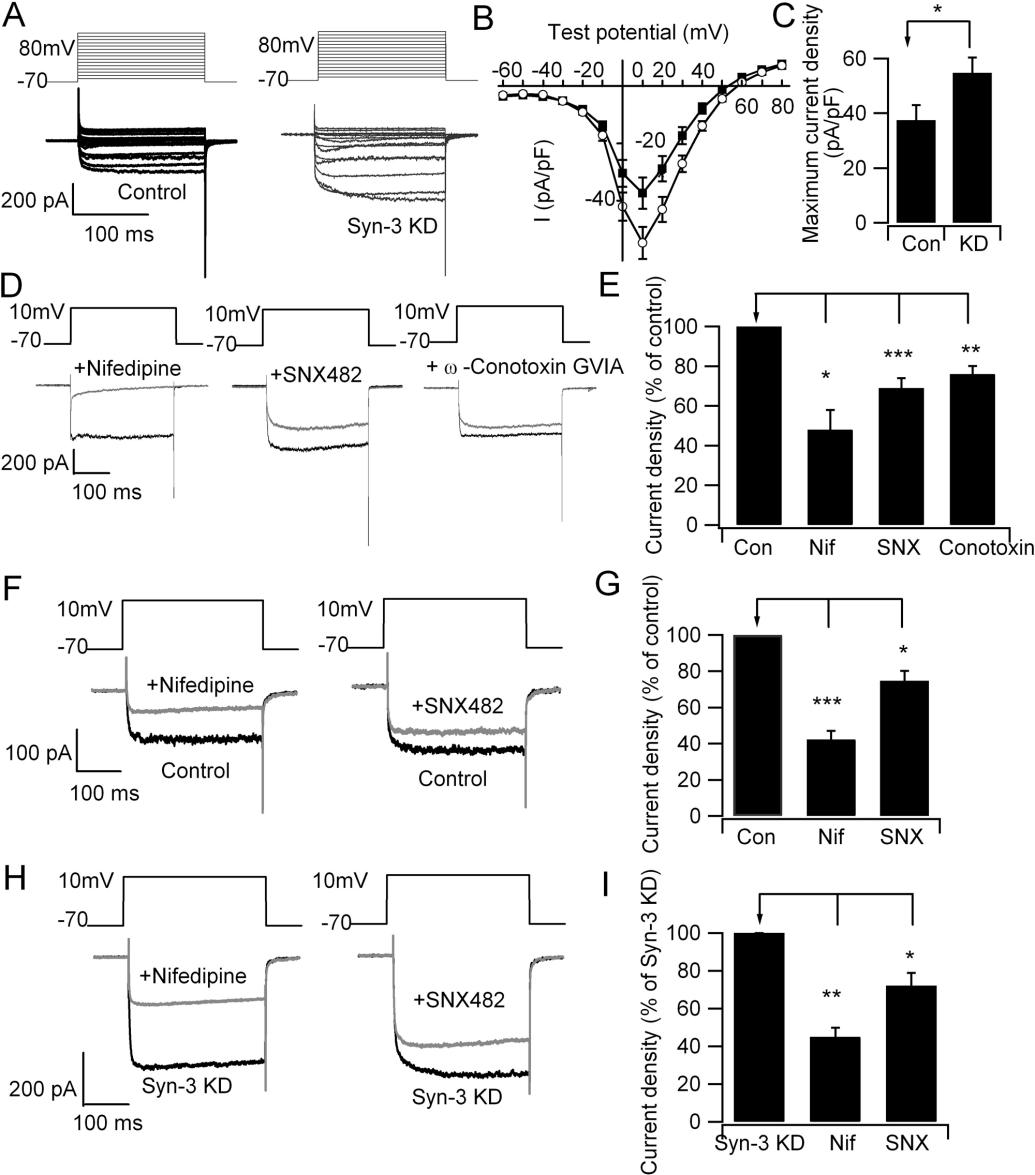
Depletion of Syntaxin 3 in INS-1 cells increased voltage-gated Ca^2+^ currents. (A) Representative traces showing Ca_v_ currents recorded in whole-cell mode from control and Syn-3 KD INS-1 cells. (B) Current-voltage relationship of Ca_v_s from control (n = 16) and Syn-3 KD (n = 11) INS-1 cells. Currents were normalized to cell capacitance to yield current density. Values are means±SEM. (C) Bar chart shows the maximum increase in current density under stimulation of 10 mV voltage. *p<0.05 for control vs Syn-3 KD (D) Representative Ca_v_ currents from normal INS-1 cells before and after treatment with nifedipine (10 μM Nif; n = 8), SNX482 (400 nM SNX; n = 9) or ω-Conotoxin GVIA (100 nM Conotoxin; n = 10); their summary analysis (E) of the maximum increase in current densities normalized to the percentage of control (Con). ***p<0.001, **p<0.01 and *p<0.05 compared to control. We then performed another set of experiments (different from A-E) to compare the effects of nifedipine and SNX on Syn-3 KD (H and I) and Control INS-1 cells (F and G). (F) Representative Ca_v_ currents from control INS-1 cells before (control, n = 25) and after treatment with nifedipine (10 μM Nif) (n = 14) or SNX482 (400 nM SNX) (n = 6); their summary analysis (G) of the maximum increase in current densities normalized to the percentage of control (Con). ***p<0.001; *p<0.05 compared to Control. (H) Representative Ca_v_ currents of Syn-3 KD INS-1 cells before (Syn-3 KD, n = 11) and after treatment with nifedipine (n = 12) or SNX482 (n = 6); and their summary analysis (I) of the maximum increase in current densities normalized as percentages of the Syn-3 KD cells. *p<0.05 compared to Syn-3 KD. Here, Syn-3 KD Ca_v_ currents were 148% of Control cells, similar to A and B.

We then assessed the functional consequence of Syn-3 interactions with these Ca_v_s by depletion of endogenous Syn-3. Syn-3 siRNA plasmid co-expressing mCherry was used to transfect INS-1 832/13 cells, which effectively reduced Syn-3 protein expression by >70%, as shown in our previous report [23]. INS-1 cells expressing mCherry would be expected to exhibit near-total depletion of Syn-3, thus ideal for single cell analysis by electrophysiology. Whole cell Ca_v_ current recording of Syn-3-depleted INS-1 cells showed the Ca_v_ current amplitudes were increased by 46% (54.8±5.6 pA/pF; n = 11; Fig 3A–3C) compared to control (mCherry transfected) cells (37.4±5.5 pA/pF; p<0.05; n = 16). L- and R-type Ca_v_s have been postulated to be the major Ca_v_s in rodents to affect first- and second-phase GSIS, respectively [17–22]. N-type Ca_v_ was reported to also contribute to first-phase GSIS [36]. We thus used selective antagonists to determine the contribution of each of these Ca_v_s to the overall Ca_v_ current density in INS-1 cells (Fig 3D). Fig 3E is the summary analysis of their Ca_v_ current densities normalized to control values, showing that amounts of Ca_v_ current blocked was 52% by nifedipine (L-type antagonist) (n = 8; p<0.05), 31% (n = 10; p<0.001) by SNX482 (R-type antagonist), and only 24% by ω-Conotoxin GVIA (N-type antagonist) (n = 9; p<0.01). This suggests that more of Ca_v_ current blocked by the L- on R-type Ca_v_ antagonists, and not N-type Ca_v_ are likely attributed to the Syn-3 actions, which would be consistent with our protein-binding data (Figs 1 and 2). To confirm that L- and R-type Ca_v_s accounted for most of the increased Ca_v_ current caused by the Syn-3 KD, we performed another set of experiments with selective blockade with nifedipine and SNX482 on Syn-3 KD cells (Fig 3H and 3I) and Control cells (Fig 3F and 3G). Nifedipine reduced the Ca_v_ current by 55%% (n = 12, p<0.05) in Syn-3 KD cells which was slightly less than the 58% reduction (n = 14; p<0.001) in Control cells. SNX482 reduced the Ca_v_ current by 28% (n = 6; p<0.05) in Syn-3 KD cells which was slightly more than the 25% reduction (n = 10; p<0.05) in Control cells.

### The Functional Domain of Syn-3 that Modulates Ca_v_ Channel Activity is Different from Syn-1A

**Fig 4:**
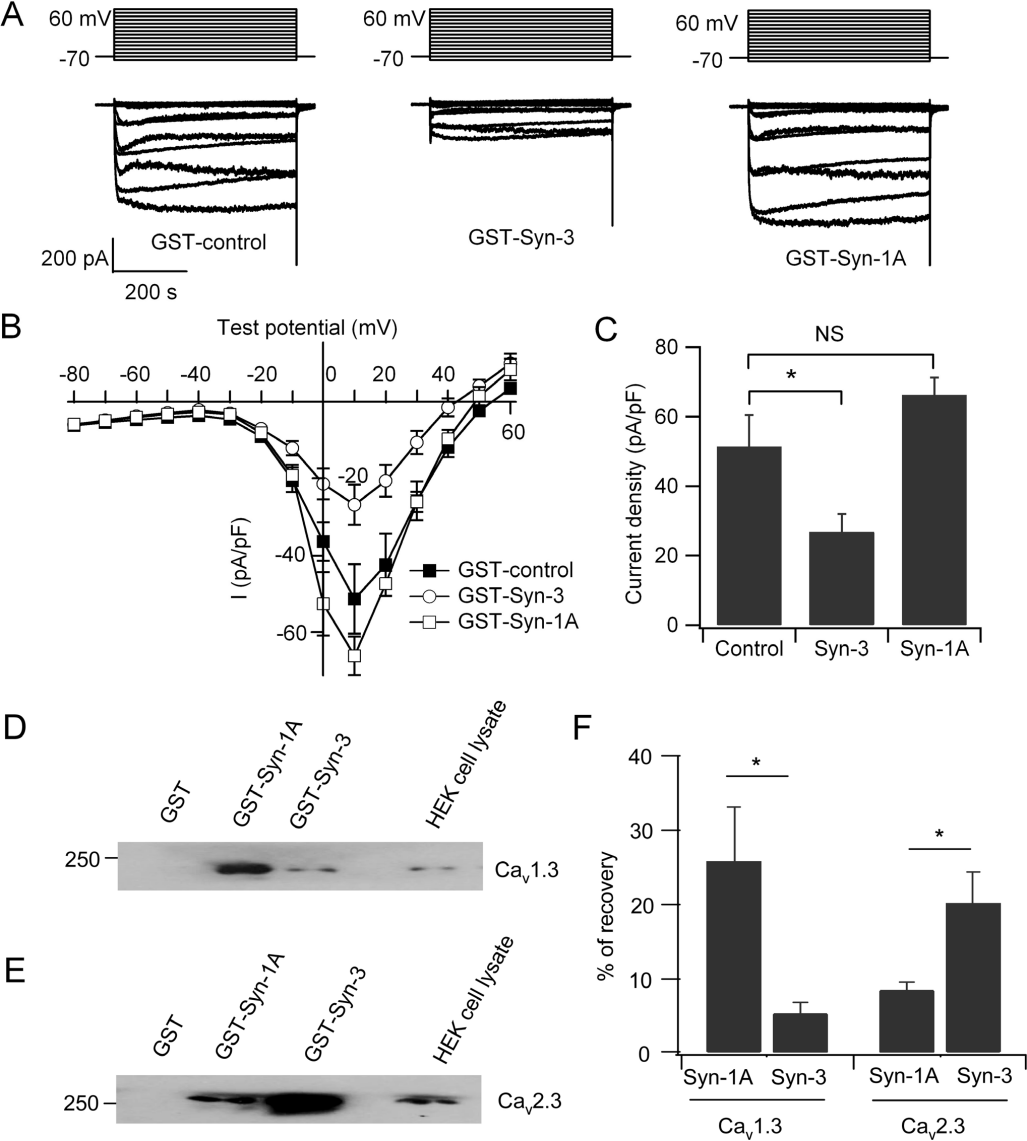
Cytoplasmic Syn-3 domain but not cytoplasmic Syn-1A domain regulates Ca_v_ currents. (A) GST-Syn-3 cytoplasmic domain (a.a. 1–263) or GST-Syn-1A cytoplasmic domain (a.a. 1–265) or GST (control) was dialyzed into INS-1 cells, then Ca_v_ currents recorded. Shown are representative traces in the whole-cell mode with stimulation from −80–60 mV. (B) Current-voltage relationship of Ca_v_ channels. Currents were normalized to cell capacitance to yield current density. (C) Bar chart showing the maximum current density in INS-1 cells dialyzed with GST control (n = 9), GST-Syn-3 (n = 9), or GST-Syn-1A (n = 8). Values are means±SEM; *p<0.05; NS: no significant difference. (D and E) Representative blots show HEK293 cells transfected with Ca_v_1.3 (D) or Ca_v_2.3 (E) subjected to pull down with 300 pmol of GST-Syn-1A (a.a. 1–265), GST-Syn-3 (a.a. 1–263) or GST. (F) Summary analysis of four separate experiments. Data was expressed as means ± SEM; *p<0.05.

It has been well studied that the putative domain of Syn-1A that modulates Ca_v_ activity is the transmembrane domain (amino acid 266–288), particularly the two vicinal cysteines (C271 and C272) [26–28]. Indeed, dialysis of the cytoplasmic domain of Syn-1A, GST-Syn-1A (a.a. 1–265), by patch pipette into INS-1 cells had no significant effects on the Ca_v_ current (66.2±5.1 pA/pF; n = 8) compared to GST control (51.4±9 pA/pF; n = 9; Fig 4A–4C). Peculiarly, the two vicinal cysteines in the Syn-1A transmembrane domain are not conserved in the transmembrane domain of Syn-3. Remarkably, dialysis of the cytoplasmic domain of Syn-3, GST-Syn-3 (a.a. 1–263) inhibited Ca_v_ current by 48% compared to control cells (26.8±5.2 pA/pF; n = 9; p<0.05; Fig 4A–4C). This result suggests that the Ca_v_-interacting domains of Syn-3 and Syn-1A are different. Syn-3 shares a low 64% amino acid identity to Syn-1A [37], suggesting that it may be the cytoplasmic domains in Syn-3 distinct from Syn-1A that bind the Ca_v_s.

We next employed protein binding and pull down assays of the cytoplasmic domains of Syn-1A (a.a. 1–265) or Syn-3 (a.a. 1–263) with Ca_v_1.3 or Ca_v_2.3 α1 subunit expressed in HEK293 cells. As shown in Fig 4D–4F with transfected HEK293 cells, GST-Syn-1A (a.a. 1–265) preferentially binds to Ca_v_1.3 with an average of 25.7±7.3% versus GST-Syn-3 (a.a. 1–263) of 5.3±1.5%, which is 4.8 times higher (Fig 4D and 4F; n = 4; p<0.05). In contrast, GST-Syn-3 binds more Ca_v_2.3 (20.1±4.1%) than GST-Syn-1A (8.4±1.0%), which is 2.4 times higher (Fig 4E and 4F; n = 4; p<0.05). These results indicate the following. First, whereas Syn-1A cytoplasmic domain can bind Ca_v_α1 subunits, preferentially L-type Ca_v_s, it seems this binding does not significantly regulate the Ca_v_ activity. Second, the cytoplasmic domain of Syn-3 binds the Ca_v_α1 subunits, preferring R-type Ca_v_2.3, thus presumably influencing Ca_v_2.3 activity. More work will be required to elucidate the putative functional Ca_v_-binding domain(s) within the Syn-3 cytoplasmic domain.

## Discussion

Taken together, these results demonstrate that Syn-3, via its cytoplasmic domain, preferentially binds and regulates R-type/Ca_v_2.3 α1 subunit. This is consistent with Syn-3’s role in mediating fusion of newcomer SGs that account for all of second-phase GSIS [23] and Ca_v_2.3’s role in mediating second-phase GSIS in rodents [21]. In contrast, Syn-1A preferentially binds L-type Ca_v_1.2 and Ca_v_1.3 to direct the PM sites of Ca^2+^ influx to where fusion of predocked SGs would occur during first-phase GSIS. Nonetheless, both syntaxins are promiscuous in binding both L- and R-type Ca_v_s and could therefore potentially assist each other in regulating various Ca_v_s perhaps when either syntaxin becomes deficient. In pancreatic islets of human type-2 diabetes, Syn-1A levels are severely reduced [38] which may presumably affect β-cell L-type Ca_v_ function that may contribute to the reduced efficiency of exocytosis of predocked insulin SGs, with ensuing reduction to absent first phase GSIS [5]. An increased in Syn-3 expression might compensate for the Syn-1A deficiency, perhaps by forming complexes with L-type Ca_v_s which could affect an increase in newcomer SGs exocytosis known to also occur in first-phase GSIS [25]. This could rescue the reduced first-phase secretion in type-2 diabetes islets that has been attributed to a loss of fusion competence of predocked SGs [38].

Syn-3 on insulin SGs [23] likely acts to direct the recruitment of newcomer SGs to PM-bound Ca_v_2.3 by forming an excitosome complex, mimicking the actions of Syn-1A-Ca_v_1.2 and Syn-1A-Ca_v_1.3 excitosomes on predocked SGs [6, 7]. During exocytosis, we postulate that Syn-3 would dissociate from Ca_v_2.3, relieving the inhibition and allowing Ca^2+^ influx to affect fusion of the newcomer SG. Whereas both syntaxins bind these Ca_v_s, the putative functional domain of Syn-1A is its transmembrane domain [26, 28] whereas the putative functional Ca_v_-binding domain of Syn-3 is within its cytoplasmic domain. It is also possible that the syntaxin-binding domain(s) in Ca_v_2.3 [34] may be different from that reported for L-type Ca_v_s [6, 11], called the synprint site localized to the cytosolic II-III linker connecting the second and third transmembrane domains. Much further work is required to determine the putative interacting domains between Syn-3 and Ca_v_2.3.

Lastly, our data are consistent with newcomer SGs (containing Syn-3) being located further away from the PM-bound R-type Ca_v_2.3 but are recruited to Ca_v_2.3 upon stimulation where they undergo minimal docking time at the PM before fusion. The latter would indicate more rapid priming and a high-affinity Ca^2+^ sensor (i.e. synaptotagmins) for newcomer SGs [39]. Synaptotagmin 7 has been purported to be the major Ca^2+^ sensor for β-cells [40], but whether this synaptotagmin or another synaptotagmin is the Ca^2+^ sensor for newcomer SGs remain to be elucidated. Consistent with this thinking, R-type Ca_v_ channel has been shown to recruit synaptotagmins to the PM to form part of the excitosome [34]. We hope that this work will trigger more future study that will lead to the full characterization of the newcomer SG excitosome as has been worked out for the predocked SG excitosome. This is of broad importance to endocrine secretory biology, as newcomer SGs likely also account for the sustained phase of secretion in other endocrine cells. While our work with the INS-1 cell line establishes proof of concept of novel Syn-3-Ca_v_ complexes influencing Ca_v_ activity, human β-cells do not contain R-type Ca_v_, but rather employ P/Q-type Ca_v_ (Ca_v_2.1) to likely mediate second-phase GSIS and consequently newcomer SG exocytosis [41]. Therefore, more exciting work will be required to assess if Syn-3 might similarly form complexes with human P/Q-type Ca_v_ (Ca_v_2.1) to mediate exocytosis of newcomer SGs in human β-cells.

## Supporting Information

S1 Fig The corresponding input proteins with the Syn-3 or Syn-1A IP experiments in Fig 1 for control and stimulated INS-1 cells. (TIF)

## Abbreviations

Syn: syntaxin
SG: insulin-containing secretory granule
GSIS: glucose-stimulated insulin secretion
PM: plasma membrane
Ca_v_: voltage-gated calcium channels
siRNA: small interfering RNA
KD: knockdown
SNARE: soluble *N*-ethylmaleimide-sensitive factor attachment protein receptor
VAMP: vesicle-associated membrane proteins
SNAP25/23: synaptosomal-associated proteins of 25/23 kDa
Syt: synaptotagmin
RRP: readily releasable pool
t-: target-
GLP-1: glucagon-like peptide-1
GST: glutathione *S*-transferase

## References

[1] Pfeffer SR. A prize for membrane magic. Cell. 2013; 155(6):1203–6. 10.1016/j.cell.2013.11.014 24315088 PMC4712707

[2] Sudhof TC, Rothman JE. Membrane fusion: grappling with SNARE and SM proteins. Science. 2009; 323(5913):474–7. 10.1126/science.1161748 19164740 PMC3736821

[3] Wheeler MB, Sheu L, Ghai M, Bouquillon A, Grondin G, Weller U, et al Characterization of SNARE protein expression in cell lines and pancreatic islets. Endocrinology. 1996; 137(4):1340–8. 8625909 10.1210/endo.137.4.8625909

[4] Huang XH, Pasyk EA, Kang YH, Sheu L, Wheeler MB, Trimble WS, et al Ca^2+^ influx and cAMP elevation overcame botulinum toxin A but not tetanus toxin inhibition of insulin exocytosis. Am J Physiol Cell Physiol. 2001; 281(3):C740–C750. 11502551 10.1152/ajpcell.2001.281.3.C740

[5] Ohara-Imaizumi M, Fujiwara T, Nakamichi Y, Okamura T, Akimoto Y, Kawai J, et al Imaging analysis reveals mechanistic differences between first- and second- phase insulin exocytosis. J Cell Biol. 2007; 177(4):695–705. 17502420 10.1083/jcb.200608132 PMC2064214

[6] Wiser O, Trus M, Hernandez A, Renstrom E, Barg S, Rorsman P, et al The voltage sensitive Lc-type Ca^2+^ channel is functionally coupled to the exocytotic machinery. Proc Natl Acad Sci U S A. 1999; 96(1):248–253. 9874804 10.1073/pnas.96.1.248 PMC15125

[7] Yang SN, Larsson O, Bränström R, Bertorello AM, Leibiger B, Leibiger IB, et al Syntaxin 1 interacts with the L(D) Subtype of voltage-gated Ca^2+^ channels in pancreatic beta cells. Proc Natl Acad Sci U S A. 1999; 96(18):10164–9. 10468580 10.1073/pnas.96.18.10164 PMC17860

[8] Ji J, Yang SN, Huang X, Li X, Sheu L, Diamant N, et al Modulation of L-type calcium channels by distinct domains within SNAP-25. Diabetes. 2002; 51(5): 1425–36. 11978639 10.2337/diabetes.51.5.1425

[9] Kang Y, Huang X, Pasyk EA, Ji J, Holz GG, Wheeler MB, et al Syntaxins-3 and –1A inhibit L-type calcium channel activity, insulin biosynthesis and exocytosis in beta-cell lines. Diabetologia. 2002; 45(2):231–41. 11935155 10.1007/s00125-001-0718-0 PMC2970522

[10] Sheng ZH, Rettig J, Cook T, Catterall WA. Calcium-dependent interaction of N-type calcium channels with the synaptic core complex. Nature. 1996; 379(6564):451–4. 8559250 10.1038/379451a0

[11] Atlas D. Functional and physical coupling of voltage-sensitive calcium channels with exocytotic proteins: ramifications for the secretion mechanism. J Neurochem. 2001; 77(4):972–85. 11359862 10.1046/j.1471-4159.2001.00347.x

[12] Atlas D. The voltage-gated calcium channel functions as the molecular switch of synaptic transmission. Annu Rev Biochem. 2013; 82:607–35. 10.1146/annurev-biochem-080411-121438 23331239

[13] Yang SN, Berggren PO. The role of voltage-gated calcium channels in pancreatic beta-cell physiology and pathophysiology. Endocr Rev. 2006; 27(6):621–76. 16868246 10.1210/er.2005-0888

[14] Dolphin AC. Calcium channel diversity: multiple roles of calcium channel subunits. Curr Opin Neurobiol. 2009; 19(3):237–44. 10.1016/j.conb.2009.06.006 19559597

[15] Nitert MD, Nagorny CL, Wendt A, Eliasson L, Mulder H. Ca_v_1.2 rather than Ca_v_1.3 is coupled to glucose-stimulated insulin secretion in INS-1 832/13 cells. J Mol Endocrinol. 2008; 41(1):1–11. 10.1677/JME-07-0133 18562674

[16] Reinbothe TM, Alkayyali S, Ahlqvist E, Tuomi T, Isomaa B, Lyssenko V, et al The human L-type calcium channel Ca_v_1.3 regulates insulin release and polymorphisms in CACNA1D associate with type 2 diabetes. Diabetologia. 2013; 56(2):340–9. 10.1007/s00125-012-2758-z 23229155

[17] Davalli AM, Biancardi E, Pollo A, Socci C, Pontiroli AE, Pozza G, et al Dihydropyridine-sensitive and -insensitive voltage-operated calcium channels participate in the control of glucose-induced insulin release from human pancreatic beta cells. J Endocrinol. 1996; 150(2):195–203. 8869586 10.1677/joe.0.1500195

[18] Barg S, Ma X, Eliasson L, Galvanovskis J, Göpel SO, Obermuller S, et al Fast exocytosis with few Ca^2+^ channels in insulin-secreting mouse pancreatic B cells. Biophys J. 2001; 81(6):3308–23. 11720994 10.1016/S0006-3495(01)75964-4 PMC1301788

[19] Schulla V, Renstrom E, Feil R, Feil S, Franklin I, Gjinovci A, et al Impaired insulin secretion and glucose tolerance in beta cell-selective Ca_v_1.2 Ca^2+^ channel null mice. EMBO J. 2003; 22(15):3844–54. 12881419 10.1093/emboj/cdg389 PMC169062

[20] Trus M, Corkey RF, Nesher R, Richard AM, Deeney JT, Corkey BE, et al The L-type voltage-gated Ca^2+^ channel is the Ca^2+^ sensor protein of stimulus-secretion coupling in pancreatic beta cells. Biochemistry. 2007; 46(50):14461–7. 18027971 10.1021/bi7016816

[21] Jing X, Li DQ, Olofsson CS, Salehi A, Surve VV, Caballero J, et al Ca_v_2.3 calcium channels control second-phase insulin release. J Clin Invest. 2005; 115(1):146–54. 15630454 10.1172/JCI22518 PMC539196

[22] Yang SN, Berggren PO. Ca_v_2.3 channel and PKClambda: new players in insulin secretion. J Clin Invest. 2005; 115(1):16–20. 15630435 10.1172/JCI23970 PMC539207

[23] Zhu D, Koo E, Kwan E, Kang Y, Park S, Xie H, et al Syntaxin-3 regulates newcomer insulin granules and compound fusion. Diabetologia. 2013; 56(2):359–69. 10.1007/s00125-012-2757-0 23132338

[24] Xie L, Zhu D, Dolai S, Liang T, Qin T, Kang Y, et al Syntaxin-4 mediates exocytosis of pre-docked and newcomer insulin granules underlying biphasic glucose-stimulated insulin secretion in human pancreatic beta cells. Diabetologia. 2015; 58(6):1250–9. 10.1007/s00125-015-3545-4 25762204

[25] Gaisano HY. Here come the newcomer granules, better late than never. Trends Endocrinol Metab. 2014; 25(8):381–8. 10.1016/j.tem.2014.03.005 24746186

[26] Trus M, Wiser O, Goodnough MC, Atlas D. The transmembrane domain of syntaxin 1A negatively regulates voltage-sensitive Ca^2+^ channels. Neuroscience. 2001; 104(2):599–607. 11377859 10.1016/s0306-4522(01)00083-5

[27] Arien H, Wiser O, Arkin IT, Leonov H, Atlas D. Syntaxin 1A modulates the voltage-gated L-type calcium channel (Ca_v_1.2) in a Cooperative Manner. J Biol Chem. 2003; 278(31):29231–9. 12721298 10.1074/jbc.M301401200

[28] Cohen R, Marom M, Atlas D. Depolarization-evoked secretion requires two vicinal transmembrane cysteines of syntaxin 1A. PLoS one. 2007; 2(12):e1273 18060067 10.1371/journal.pone.0001273 PMC2094736

[29] Xie L, Zhu D, Kang Y, Liang T, He Y, Gaisano HY. Exocyst sec5 regulates exocytosis of newcomer insulin granules underlying biphasic insulin secretion. PLoS One. 2013; 8(7):e67561 10.1371/journal.pone.0067561 23844030 PMC3699660

[30] Zhu D, Zhang Y, Lam PP, Dolai S, Liu Y, Cai EP, et al Dual Role of VAMP8 in Regulating Insulin Exocytosis and Islet beta Cell Growth. Cell Metab. 2012; 16(2):238–49. 10.1016/j.cmet.2012.07.001 22841572

[31] Hohmeier HE, Newgard CB. Cell lines derived from pancreatic islets. Mol Cell Endocrinol. 2004; 228(1–2):121–8. 15541576 10.1016/j.mce.2004.04.017

[32] Lam PP, Ohno M, Dolai S, He Y, Qin T, Liang T, et al Munc18b is a major mediator of insulin exocytosis in rat pancreatic β-cells. Diabetes. 2013; 62(7):2416–28. 10.2337/db12-1380 23423569 PMC3712044

[33] Kang Y, Leung YM, Manning-Fox JE, Xia F, Xie H, Sheu L, et al Syntaxin-1A inhibits cardiac K_ATP_ channels by its actions on nucleotide binding folds 1 and 2 of sulfonylurea receptor 2A. J Biol Chem. 2004; 279(45):47125–31. 15339904 10.1074/jbc.M404954200

[34] Cohen R, Atlas D. R-type voltage-gated Ca^2+^ channel interacts with synaptic proteins and recruits synaptotagmin to the plasma membrane of Xenopus oocytes. Neuroscience. 2004; 128(4):831–41. 15464290 10.1016/j.neuroscience.2004.07.027

[35] Sheng ZH, Rettig J, Takahashi M, Catterall WA. Identification of a syntaxin-binding site on N-type calcium channels. Neuron. 1994; 13(6):1303–13. 7993624 10.1016/0896-6273(94)90417-0

[36] Taylor JT, Huang L, Keyser BM, Zhuang H, Clarkson CW, Li M. Role of high-voltage-activated calcium channels in glucose-regulated beta-cell calcium homeostasis and insulin release. Am J Physiol Endocrinol Metab. 2005; 289(5):E900–8. 15956052 10.1152/ajpendo.00101.2005

[37] Bennett MK, García-Arrarás JE, Elferink LA, Peterson K, Fleming AM, Hazuka CD, et al The syntaxin family of vesicular transport receptors. Cell. 1993; 74(5):863–73. 7690687 10.1016/0092-8674(93)90466-4

[38] Ostenson CG, Gaisano H, Sheu L, Tibell A, Bartfai T. Impaired gene and protein expression of exocytotic soluble N-ethylmaleimide attachment protein receptor complex proteins in pancreatic islets of type 2 diabetic patients. Diabetes. 2006; 55(2):435–40. 16443778 10.2337/diabetes.55.02.06.db04-1575

[39] Pedersen MG, Sherman A. Newcomer insulin secretory granules as a highly calcium-sensitive pool. Proc Natl Acad Sci U S A. 2009; 106(18):7432–6. 10.1073/pnas.0901202106 19372374 PMC2678592

[40] Gustavsson N, Lao Y, Maximov A, Chuang JC, Kostromina E, Repa JJ, et al Impaired insulin secretion and glucose intolerance in synaptotagmin-7 null mutant mice. Proc Natl Acad Sci U S A. 2008; 105(10):3992–7. 10.1073/pnas.0711700105 18308938 PMC2268794

[41] Braun M, Ramracheya R, Bengtsson M, Zhang Q, Karanauskaite J, Partridge C, et al Voltage-gated ion channels in human pancreatic beta-cell: electrophysiological characterization and role in insulin secretion. Diabetes. 2008; 57(6):1618–28. 10.2337/db07-0991 18390794

