# Supplementary figures and images for: Syntaxin-3 Binds and Regulates Both R- and L-Type Calcium Channels in Insulin-Secreting INS-1 832/13 Cells
Source: PLoS One. 2016 Feb 5;11(2):e0147862. doi: 10.1371/journal.pone.0147862 (PMC4743851; doi:10.1371/journal.pone.0147862)

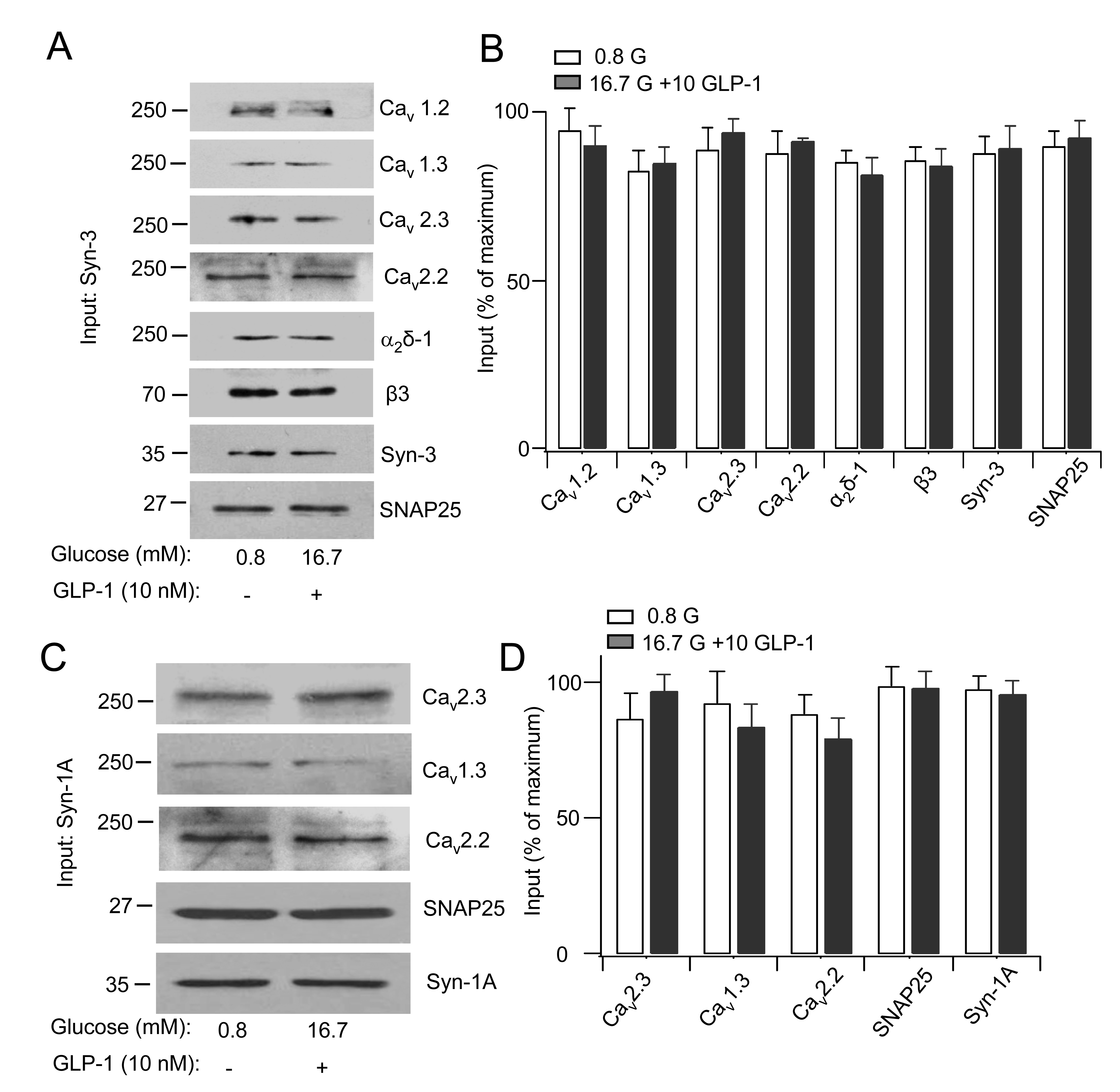

Supplement: S1 Fig — (TIF) [file pone.0147862.s001.tif]
